# Comparison of mental health indicators in clinical psychologists with the general population during the COVID-19 pandemic

**DOI:** 10.1038/s41598-023-32316-x

**Published:** 2023-03-28

**Authors:** Elke Humer, Barbara Pammer, Yvonne Schaffler, Oswald D. Kothgassner, Anna Felnhofer, Andrea Jesser, Christoph Pieh, Thomas Probst

**Affiliations:** 1 Department for Psychosomatic Medicine and Psychotherapy, University for Continuing Education Krems, Krems, Austria; 2 Department of Child and Adolescent Psychiatry, Comprehensive Center for Pediatrics, Medical University of Vienna, Vienna, Austria; 3 Department of Pediatrics and Adolescent Medicine, Division of Pediatric Pulmonology, Allergology and Endocrinology, Medical University of Vienna, Vienna, Austria

**Keywords:** Occupational health, Psychiatric disorders, Health care

## Abstract

Mental healthcare professionals face diverse challenges during the COVID-19 pandemic, which may augment their risk of experiencing adverse mental health outcomes themselves. We aimed to compare depressive, anxiety, insomnia, and stress symptoms in Austrian clinical psychologists during the COVID-19 pandemic with the Austrian general population. A total of N = 172 Austrian clinical psychologists (91.9% women; mean age: 44.90 ± 7.97 years) participated in an online survey in spring 2022. A representative sample (N = 1011) of the Austrian general population was surveyed simultaneously. Symptoms of depression (PHQ-2), anxiety (GAD-2), insomnia (ISI-2), and stress (PSS-10) were assessed. Differences in the prevalence of clinically relevant symptoms were analyzed using univariate (Chi-squared tests) and multivariable (binary logistic regression including covariates age and gender) analyses. Clinical psychologists showed lower adjusted odds for exceeding the cut-offs for clinically relevant depression (aOR 0.37), anxiety (aOR 0.50), and moderate to high stress levels (aOR 0.31) compared to the general population (*p* < 0.01). No difference was observed for insomnia (aOR 0.92; *p* = 0.79). In conclusion, clinical psychologists experience better mental health than the general population during the COVID-19 pandemic. Future studies are needed to analyze the underlying reasons.

## Introduction

The COVID-19 pandemic and the preventive measures implemented to combat it have been shown to negatively impact mental health^1,2^. Healthcare professionals are particularly challenged during the pandemic as they are particularly vulnerable to experiencing adverse mental health outcomes such as depression, anxiety, and high stress levels^3,4^. Studies on mental health in healthcare workers during the COVID-19 crisis focused mainly on physicians and nurses, while less is known about mental health in mental healthcare professionals, such as licensed clinical psychologists.

Not only during the pandemic, then rather in general, mental health in clinical psychologists is seldom the focus of empirical studies. The largest study investigating mental health issues among clinical psychologists before the COVID-19 crisis reported a higher lifetime prevalence of mental health symptoms compared to the general population (63% vs 41%)^5^. A high mental health burden in individuals providing mental healthcare does not only negatively affect the person, but is also detrimental to patient care^6^.

The pandemic went along with changes in the everyday practice of mental healthcare professionals, such as switching to remote therapeutic formats, working with face masks, dealing with waiting lists due to an increased need for psychological treatment, or changes regarding the symptoms patients are presenting^7–9^. Whether these professional challenges put clinical psychologists at a higher risk for adverse mental health outcomes than the general population has not been investigated.

A high level of resilience, i.e., the individual`s ability to cope with adverse situations^10^, is a protective factor against mental health symptoms, such as depression or anxiety^11–13^. Previous studies highlight that the ability to deal with crises varies considerably among individuals and is affected by personality factors, interpersonal as well as social variables^11^. Studies conducted during the COVID-19 pandemic revealed moderate levels of resilience among healthcare workers^11,14^, emphasizing the need to foster mental hygiene and to promote resilience among healthcare personnel during the COVID-19 pandemic^11^. The increased mental health burden in the general population observed since the emergence of the pandemic^15–17^ requires a state of high alert in mental healthcare professionals during as well as in the aftermath of the COVID-19 pandemic. Whether clinical psychologists positively adapt to this extraordinary public health crisis with psychological growth or experience psychological injury, remains unknown to date.

This study aimed to compare mental health indicators (i.e., clinically relevant depressive and anxiety symptoms, insomnia, and stress) in Austrian clinical psychologists during the COVID-19 pandemic with the Austrian general population.

## Results

## Study sample characteristics

In total, N = 172 clinical psychologists participated. They were 44.90 ± 7.97 years old, and 91.9% were female (compared to 85.1% in the list of licensed clinical psychologists). They were 13.91 ± 7.72 years in the profession (compared to 12.03 ± 6.91 years of all licensed clinical psychologists), and 74.4% worked in private practice.

The sample from the Austrian general population surveyed in April 2022 comprised N = 1011 adult individuals. They were 46.16 ± 16.89 years old, and 50.6% were female. While age differences between both samples did not reach significance (t(483.89) = 1.57; *p* = 0.12), there was a clear gender difference between both samples (χ^2^ (1) = 101.68; *p* < 0.001; Suppl. Table 1).

## Mental health indicators in clinical psychologists vs the general population

Univariate analyses (Table 1) revealed lower prevalence rates of depression, anxiety, and moderate to high stress in clinical psychologists vs the general population (*p* ≤ 0.02). At the same time, no difference in insomnia was observed between both groups (*p* = 0.87).

**Table 1:** Proportion of participants exceeding the cut-off scores for moderate depression/anxiety/insomnia and stress by group (n = 1183).

Variable		Group		*p*
		General population (n = 1011)	Clinical psychologists (n = 172)	
Depression	% n	24.4% 247	12.2% 21	χ^2^ (1) = 12.53; *p* < 0.001
Anxiety	% n	20.0% 202	12.2% 21	χ^2^ (1) = 5.80; *p* = 0.016
Insomnia	% n	9.7% 98	9.3% 16	χ^2^ (1) = 0.03; *p* = 0.87
Moderate/high stress	% n	64.4% 651	43.0% 74	χ^2^ (1) = 28.29; *p* < 0.001

*p*: *p*-values (2-tailed); χ^2^: Chi-squared-test; Depression: ≥ 3 points on the Patient Health Questionnaire 2 scale; Anxiety: ≥ 3 points on the Generalized Anxiety Disorder 2 scale; Insomnia: ≥ 6 on the 2-item Insomnia Severity Index; Moderate/High Stress: ≥ 14 points on the Perceived Stress Scale 10.

**Figure 1:**
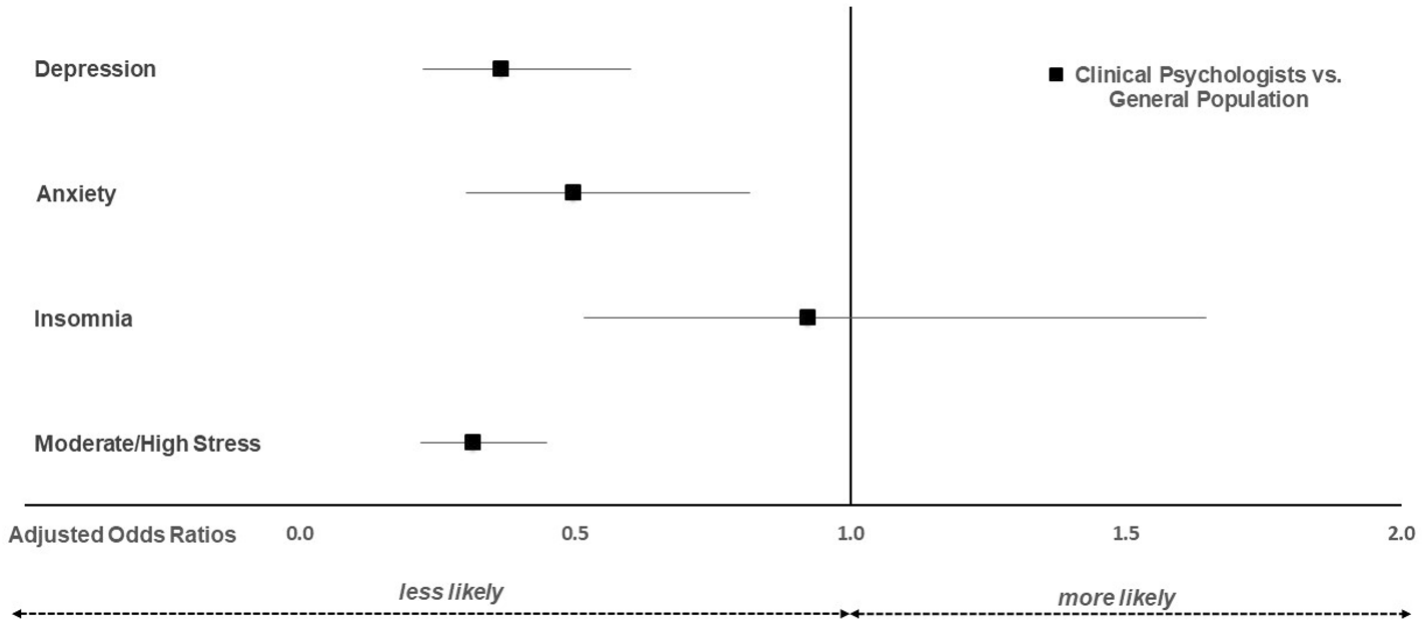
Adjusted odds ratios for clinically relevant depression, anxiety, insomnia, and stress in clinical psychologists vs the general population.

Multivariable analyses adjusting for age and gender confirmed these findings. As depicted in Fig. 1, clinical psychologists, compared to the general population, were less likely to experience clinically relevant depression (aOR 0.37; *p* < 0.001), anxiety (aOR 0.50; *p* = 0.006), and moderate to high-stress levels (aOR 0.31; *p* < 0.001), whereas no difference for insomnia was observed (aOR 0.92; *p* = 0.79).

Male gender decreased the odds for depression (aOR 0.66; 95% CI 0.49, 0.89; *P* = 0.006), anxiety (aOR 0.71; 95% CI 0.52, 0.97; *P* = 0.031), and moderate/high stress (aOR 0.67; 95% CI 0.52, 0.87; *P* = 0.003), whereas for insomnia no significant difference was observed (aOR 0.93; 95% CI 0.62, 1.40; *P* = 0.72). With increasing age, the odds for depression (aOR 0.97; 95% CI 0.96, 0.98; *P* < 0.001), anxiety (aOR 0.97; 95% CI 0.96, 0.98; *P* < 0.001), and moderate/high stress (aOR 0.96; 95% CI 0.95, 0.97; *P* < 0.001) decreased. No association of age with the odds for insomnia was found (aOR 0.99; 95% CI 0.98, 1.00; *P* = 0.14).

As the clinical psychologists’ sample comprised mainly of women (91.9%), all analyses were also conducted including only female participants from both groups. Univariate analyses on female participants (Suppl. Table 2) confirmed the findings observed for the total sample. Female clinical psychologists had lower prevalence rates of depression, anxiety, and moderate to high stress vs the female general population (*p* ≤ 0.004). No difference in female participants exceeding the cut-off for clinically relevant insomnia was observed between both groups (*p* = 0.63).

Multivariable analyses adjusting for age including only female participants also replicated the findings from the total sample. As depicted in Suppl. Figure 1, clinical psychologists, compared to the general population showed lower adjusted odds for exceeding the cut-offs for depression (aOR 0.37; *p* < 0.001), anxiety (aOR 0.48; *p* = 0.006), and moderate to high-stress levels (aOR 0.28; *p* < 0.001), whereas no difference for insomnia was observed (aOR 0.86; *p* = 0.63). With increasing age, the odds for depression (aOR 0.98; 95% CI 0.97, 0.99; *P* < 0.001), anxiety (aOR 0.97; 95% CI 0.96, 0.98; *P* < 0.001), and moderate to high stress (aOR 0.96; 95% CI 0.95, 0.97; *P* < 0.001) decreased in women. No association of age with the odds for insomnia was found in women (aOR 1.00; 95% CI 0.98, 1.01; *P* = 0.57).

## Discussion

In Austria the COVID-19 pandemic went along with an increased demand for mental healthcare services^9,18^ which was sustained even during spring 2022 when most pandemic-related measures were lifted^19^. A good mental health status of clinical psychologists is essential for their ability to deliver optimal psychological care^20^. Therefore, knowledge of the mental health status of clinical psychologists in times of the pandemic is essential to reveal whether measures should be taken to foster mental health in clinical psychologists. To our knowledge, this is the first study comparing the mental health of clinical psychologists with the general population during the COVID-19 pandemic. Results suggest that clinical psychologists show lower levels of mental health symptom severity than a representative sample of the general population after two years of COVID-19.

The reasons explaining these findings are undoubtedly manifold and cannot be conclusively clarified with this study. Potential reasons for the better mental health status of clinical psychologists as compared to the general population during the pandemic might be a better socioeconomic status of clinical psychologists that may have some protective function^21,22^. Multivariable analyses investigating the independent contribution of several sociodemographic factors (i.e., age, gender, education, income, employment status, partnership status, migration background) with mental health indicators carried out on the sample of the Austrian general population at hand, revealed that next to young age, low income was the main risk factor for poor mental health^15^. Therefore, it can be speculated that fewer economic challenges in clinical psychologists vs the general population contributed to the observed differences in mental health. It is also possible that clinical psychologists are more accustomed to handling stressful situations due to their work with mentally distressed individuals and can therefore recruit more resilience factors during an acute stressor such as a public health emergency. It seems likely that clinical psychologists practice more adaptive coping strategies and self-care such as keeping a daily balance between the demands, structuring the working day, and keeping a balance between work and private life^23^. Another potential reason might be the high rate of clinical psychologists working in private practice, which allows them more autonomy and likely increases their satisfaction with their work^24,25^. Overall, future studies are needed to analyze the underlying reasons for the better mental health status of clinical psychologists compared to the general population during the COVID-19 pandemic.

Limitations are the cross-sectional design, the critical missing co-variables, such as income, a potential selection bias due to the online nature of the study, a potential response bias due to clinical psychologists likely being familiar with the mental health questionnaires applied, as well as the missing validation of self-rated symptoms by clinical interviews. Furthermore, the study was limited to the Austrian population and thus results might not generalize to other countries as mental health care systems as well as pandemic-related factors vary considerably between countries.

## Methods

## Design

An online survey among licensed Austrian clinical psychologists was conducted between April 11 and May 31, 2022. The link to the survey was sent via e-mail to all clinical psychologists registered in the Austrian Federal Ministry of Social Affairs, Health, Care and Consumer Protection list, providing a valid e-mail address. Participation was voluntary, without incentives. In Austria, clinical psychologists are psychologists (at least a diploma/master’s degree) who also obtained postgraduate training in clinical psychology.

A representative sample of the Austrian general population according to age, gender, region, and educational level was recruited between April 19 and 26, 2022, through the Marketagent.com online research GmbH panel (Baden, Austria; certified under ISO 20252). Respondents were recruited by quota sampling by the Marketagent project team who organized and coordinated data collection.

This study was conducted following the Declaration of Helsinki and approved by the Ethics Committee of the University for Continuing Education Krems, Austria (Ethical numbers: EK GZ 26/2018-2021, EK GZ 11/2021-2024). All participants gave electronic informed consent to participate and complete the questionnaires.

## COVID-19 situation during the study

The COVID-19 situation in Austria was characterized by rather strict policies, including four nationwide strict lockdowns during the first two years of the pandemic^15^. The last nationwide lockdown ended in December 2021, while a lockdown for unvaccinated people remained in place until the end of January 2022^26^. The emergence of the Omicron variant around the turn of the year 2021/2022 went along with new highs in daily confirmed COVID-19 cases, but no congestion of intensive care unit facilities due to the milder course of the disease^26,27^. In April 2022 infection numbers declined^27^ and most protective measures were lifted. Protective measures in place during the time of the study included face mask mandates in essential shops, public transport, nursing homes, and hospitals, as well as the need to prove that someone is vaccinated, recently recovered from COVID-19, or tested negative for SARS-CoV-2 upon entering Austria^26^. During this time, the job market was characterized by a steady decline in unemployment rates and a high number of vacancies in the labor market^28^. While the unemployment rate in spring 2022 was even lower compared to pre-pandemic times^29^, inflation soared to a multidecade high, causing the most financial distress in persons with low income^30,31^.

## Measures

Depressive symptoms were assessed with the two-items version of the Patient Health Questionnaire^32^. The PHQ-2 yields a total score from 0 to 6, with a cut-off point of ≥ 3 being suggested to be indicative of clinically relevant depressive symptoms^33^. Cronbach's alpha was α = 0.71 in the present clinical psychologists' sample and α = 0.77 in the general population sample.

Anxiety symptoms were assessed with the short version of the Generalized Anxiety Disorder scale^34,35^. The GAD-2 measures feelings of nervousness, anxiety, or being on edge, as well as the inability to stop or control worrying with two items yielding a total scale from 0 to 6. Scores of ≥ 3 have been defined to indicate clinically relevant anxiety symptoms^35^. Cronbach's alpha was α = 0.69 in the sample of clinical psychologists and α = 0.81 in the general population.

Sleep quality was evaluated with the two items version of the Insomnia Severity Index (ISI)^36^. The total scores of the ISI-2 range from 0 to 8. A cut-off score of ≥ 6 has been recommended to define insomnia disorder^37^. Cronbach's alpha was α = 0.77 in the clinical psychologists and α = 0.75 in the general population.

The Perceived Stress Scale (PSS-10) measured subjective stress levels. The total scores range from 0 to 40, with a cut-off score of ≥ 14 indicative of moderate to high stress^38^. Cronbach's alpha was α = 0.89 in the clinical psychologists' sample and α = 0.85 in the general population sample.

## Statistical analyses

Descriptive statistics were conducted to describe sociodemographic characteristics. Chi-squared tests and T-tests for independent samples were applied to assess differences in sociodemographic characteristics between participating clinical psychologists and the representative sample of the Austrian general population.

Univariate analyses were computed using Chi-squared tests to assess differences in the prevalence of mental health indicators (moderate depression, anxiety, insomnia, and stress) between clinical psychologists and the general population.

Multivariable binary logistic regressions were applied to account for potential confounders between both groups (clinical psychologists vs general population) by including covariates age and gender. To assess the statistical uncertainty, adjusted odds ratios (aOR) and their 95% confidence intervals (CIs) were computed.

As the clinical psychologists' sample comprised mainly of women and significant gender differences in mental health indicators have been reported previously^16,17^, all analyses were carried out for the total sample and the female sample separately.

Analyses were conducted in SPSS version 26 (IBM Corp, Armonk, NY, USA), with *p*-values < 0.05 being considered statistically significant (2-sided tests).

## Supplementary Information

Supplementary Table S1.

Supplementary Table S2.

Supplementary Figure S1.

## Data Availability

The datasets analyzed during the current study are available from the corresponding author (Elke Humer, elke.humer@donau-uni.ac.at) upon reasonable request.
