# Supplementary material for: Comparison of mental health indicators in clinical psychologists with the general population during the COVID-19 pandemic
Source: Sci Rep. 2023 Mar 28;13:5050. doi: 10.1038/s41598-023-32316-x (PMC10043835; doi:10.1038/s41598-023-32316-x)
Supplement: Supplementary file 1 — Supplementary Table S1. [file 41598_2023_32316_MOESM1_ESM.pdf]

Suppl. Table 1 Study sample characteristics

	Participating clinical psychologists (n = 172)	General population (n = 1,011)	Statistics
**Gender**			
Female, % (N)	91.9% (158)	50.6% (512)	$\chi 2$ (1) = 101.68;
Male, % (N)	8.1% (14)	49.4% (499)	$P < 0.001$
**Age in years, M (SD)**	44.90 (7.97)	46.16 (16.89)	t(483.89) = 1.57; $P = 0.12$
**Education**			
No school education, % (N)	0	0.6% (6)	$\chi 2$ (5) = 608.58;
Secondary school, % (N)	0	19.5% (197)	$P < 0.001$
Apprenticeship, % (N)	0	34.5% (349)	
Vocational secondary school, % (N)	0	16.7% (169)	
High School	0	16.6% (168)	
University	100% (172)	12.1% (122)	
**Region**			
Vienna	25.6% (44)	19.2% (194)	$\chi 2$ (8) = 6.54;
Upper Austria	14.5% (25)	16.0% (162)	$P = 0.59$
Lower Austria	17.4% (30)	21.8% (220)	
Carinthia	7.0% (12)	5.9% (60)	
Styria	15.7% (27)	14.6% (148)	
Tyrol	7.0% (12)	8.4% (85)	
Salzburg	7.0% (12)	6.4% (65)	
Burgenland	1.7% (3)	3.4% (34)	
Vorarlberg	4.1 % (7)	4.3% (43)	
