# Supplementary material for: Comparison of mental health indicators in clinical psychologists with the general population during the COVID-19 pandemic
Source: Sci Rep. 2023 Mar 28;13:5050. doi: 10.1038/s41598-023-32316-x (PMC10043835; doi:10.1038/s41598-023-32316-x)
Supplement: Supplementary file 2 — Supplementary Table S2. [file 41598_2023_32316_MOESM2_ESM.docx]

**Suppl. Table 2** Proportion of female participants exceeding the cut-off scores for moderate depression/anxiety/insomnia and stress by group (n = 670)

|  | |  | Group | | *p* |
| --- | --- | --- | --- | --- | --- |
| Variable | |  | General population (n = 512) | Clinical psychologists  (n = 158) |  |
| Depression | %  n | | 27.9% 143 | 12.7% 20 | χ^2^ (1) = 15.30;  *p* < 0.001 |
| Anxiety | %  n | | 22.7% 116 | 12.0% 19 | χ^2^ (1) = 8.48;  *p* = 0.004 |
| Insomnia | % n | | 10.2% 52 | 8.9% 14 | χ^2^ (1) = 0.23;  *p* = 0.633 |
| Moderate/High Stress | % n | | 69.1% 354 | 41.1% 65 | χ^2^ (1) = 40.41;  *p* < 0.001 |

*p*: *p*-values (2-tailed); χ^2^: Chi-squared-test; Depression: ≥ 3 points on the Patient Health Questionnaire 2 scale; Anxiety: ≥ 3 points on the Generalized Anxiety Disorder 2 scale; Insomnia: ≥ 6 on the 2-item Insomnia Severity Index; Moderate/High Stress: ≥ 14 points on the Perceived Stress Scale 10.
