# Supplementary material for: Comparison of mental health indicators in clinical psychologists with the general population during the COVID-19 pandemic
Source: Sci Rep. 2023 Mar 28;13:5050. doi: 10.1038/s41598-023-32316-x (PMC10043835; doi:10.1038/s41598-023-32316-x)
Supplement: Supplementary file 3 — Supplementary Figure S1. [file 41598_2023_32316_MOESM3_ESM.docx]

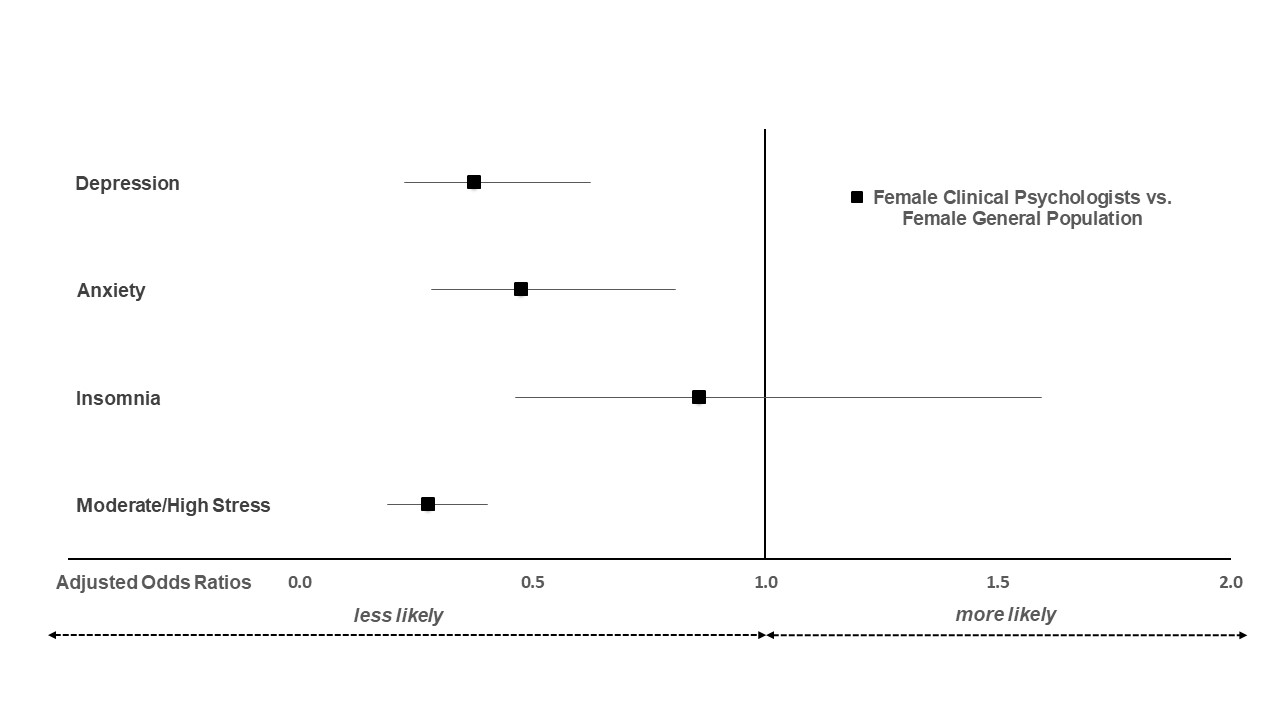


**Suppl. Figure 1.** Adjusted odds ratios for clinically relevant depression, anxiety, insomnia, and stress in female clinical psychologists vs the female general population
